# Automatic identification of angiogenesis in double stained images of liver tissue

**DOI:** 10.1186/1471-2105-10-S11-S13

**Published:** 2009-10-08

**Authors:** Mutlu Mete, Leah Hennings, Horace J Spencer, Umit Topaloglu

**Affiliations:** 1 Information Technology Research, University of Arkansas for Medical Sciences, Little Rock, Arkansas, USA; 2 Department of Pathology, University of Arkansas for Medical Sciences, Little Rock, Arkansas, USA; 3 Biostatistics, University of Arkansas for Medical Sciences, Little Rock, Arkansas, USA

## Abstract

**Background:**

To grow beyond certain size and reach oxygen and other essential nutrients, solid tumors trigger angiogenesis (neovascularization) by secreting various growth factors. Based on this fact, several researches proposed that density of newly formed vessels correlate with tumor malignancy. Vessel density is known as a true prognostic indicator for several types of cancer. However, automated quantification of angiogenesis is still in its primitive stage, and deserves more intelligent methods by taking advantages accruing from novel computer algorithms.

**Results:**

The newly introduced characteristics of subimages performed well in identification of region-of-angiogenesis. The proposed technique was tested on 522 samples collected from two high-resolution tissues. Having 0.90 overall f-measure, the results obtained with Support Vector Machines show significant agreement between automated framework and manual assessment of microvessels.

**Conclusion:**

This study introduces a new framework to identify angiogenesis to measure microvessel density (MVD) in digitalized images of liver cancer tissues. The objective is to recognize all subimages having new vessel formations. In addition to region based characteristics, a set of morphological features are proposed to differentiate positive and negative incidences.

## Introduction

As a general term, angiogenesis (neovascularization) is the formation of new blood vessels. Under normal conditions, proliferation of blood vessel occurs in the various organs as a part of development, growth, and repair of body. Similarly, tumor angiogenesis is the generation of new blood vessels around cancerous growths. To reach oxygen and other essential nutrients and remove waste products, solid tumors trigger angiogenesis by secreting various growth factors (e.g., Vascular Endothelial Growth Factor or VEGF) [1]. This fact makes MVD an interesting measure for clinical prognosis of solid tumors. In the context of this study, the term MVD refers to a quantitative measurement of blood vessels proliferation within active region of a tumor. In the last decade, a number of studies quantified MVD to research a possible correlation with the biological behavior of various types of carcinomas. In these studies, angiogenesis has been reported to be correlated with the depth of invasion, such as in colonic carcinoma [2,3], breast cancer [4], renal cell carcinoma [5], lung carcinomas [6], and squamous cell carcinomas of the esophagus [7]. However, in some very rare cancers, such as synovial sarcomas and cerebellar hemangioblastomas, no correlation is reported between microvessel density and immunohistochemical expression of vascular endothelial growth factor [8,9].

As obvious, microvessel count is a *bona fide *indicator for metastasis and prognosis; thus, histological examination of neovascularization is mandatory in selective cases. Currently, quantification of microvessels in tumor regions is assessed manually. Reliable assessments can be achieved with strict standardized conditions, i.e., certain staining amount, careful selection of hot spots (areas of high vascular density), and carefully articulated description of microvessels. At least, a mediocre reproducibility is reported by De Jong et al. in [10]. Nevertheless, tedious work is required for the review of many sections; therefore, manual counting still remains time consuming, error prone, and vulnerable to inter- and intra-observer variability [11].

One other motivation for automated and semi-automated systems is that some rural areas may not have enough pathologists to assess large number of slides; thus, observer error increases with rapid evaluation of the cases. However, for a reliable and reproducible assessment of tumor neovascularization, validation procedures and quality control protocols are obligatory. For these very reasons, automated systems, e.g., computer aided counting of microvessel and hot-spot detection, would be a significant help for histopathologists.

A few semi-automatic methods were developed particularly to avoid variability due to human error and evaluated in comparison to manual counting methods for breast cancer [11], squamous cell carcinoma of the head and neck [12], urinary bladder carcinoma [13], and acinic cell carcinoma [14]. Belien et al. [11] used fixed-thresholding *-one of the simplest methods in image segmentation *and object features in grey-scaled images of breast cancer to count microvessels in hot spots. Each experimental image is automatically digitalized and standardized manually under the microscope. Erovic et al. [12] examined agreement between computer and investigator counts for tumor samples of 50 patients with squamous cell carcinoma of the head and neck. The framework is semi-automated since hot spots of tumor specimens are selected manually. It is reported that computerized microvessel determination could be used as a reliable method for microvessel counts, which seems to be superior to manual counting. Both semi-automated and automated methods for urinary bladder carcinoma are discussed in the study of Wester et al. [13]. Experiments demonstrated that quantification in ten images, selected in a descending order of MVD by visual judgment, showed a poor observer capacity to estimate and rank MVD. Luukkaa et al. [12] employed computer-assisted analysis of CD34 immunoreactivity in acinic cell cancer (ACC), a morphologically diverse group of malignancies. Results suggest that larger vessel size, vessel irregularity, and lower intensity of CD34-positive vessel staining may indicate unfavorable prognosis.

From the technical point of view, vast majority of studies in the literature focus on a partial (not whole) image of a specimen. Image artifacts, such as shading variation due to lighting irregularities [13], vignetting by the digital camera system, heterogeneous sensitivity during imaging, and lens artifact, are inevitable issues associated with the systems running on partial images. In both, automated and manual, methods, observer subjectivity in the selection of hot spots of tumor regions causes variability in results.

**Figure 1:**
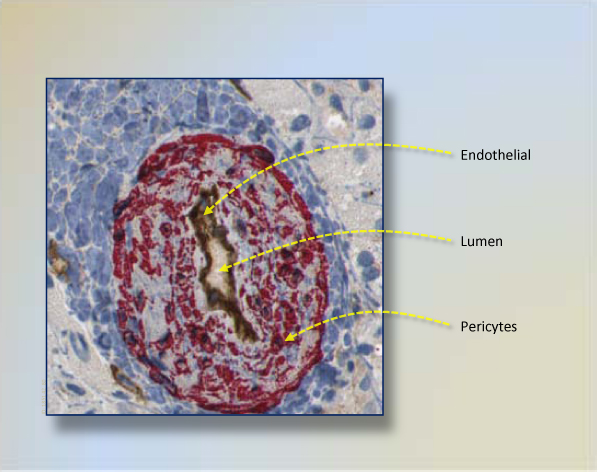
**A subimage showing cross-section of a mature vessel**. Brown stained endothelium cells border a vessel lumen that is inner section of a vessel. Pericytes are stained red and cover endothelium cells. The closeness between red stained regions and brown ones clearly indicate that red stained regions are not fibroblasts or smooth muscle cells.

In this pilot study, we introduce a framework for fully automated region-of-microvessel identification in histopathological sections of human liver carcinoma. This framework benefits from the fact that blood vessels are composed of two interacting cell types. Endothelial cells form the inner lining of the vessel wall, and perivascular cells-referred as pericytes, vascular smooth muscle cells or mural cells that envelop the surface of the vascular tube [15]. Thus, this research partially relates identification of these two components: pericytes and endothelial. These regions are shown in sample vessel cut of Figure 1. The other complementary section of framework is to quantify subimages based on known region-of-interests. Highly informative features, such as statistical, histomorphological, and fractal dimension of vessel regions, are extracted from subimages. In this way, we represent each subimage with a set of features. To find positive subimages that have angiogenesis formation *-even very small ones*, Support Vector Machines [16] classifiers are used. Selected subimages are reported as positive with associated probabilities. This study introduces very pertinent features to be utilized in angiogenesis identification; thus, serves as first phase of microvessel counting in double stained liver tissue of human. Furthermore, it is very flexible to use this framework with other types of carcinomas.

Recall that we do not use regular partial images obtained from a regular digital camera attached to a microscope. Recent improvements in imaging techniques led to discovery of virtual histological slides, where an automated light microscope [17] scans a glass slide and generates a virtual slide at a resolution of 0.25 *μ*m/pixel (1 micron (*μ*m) = 1/1,000,000 meter). Output images are of sufficiently high quality to attract immense interest within the research community. Since a virtual slide wholly covers the glass slide, and minimizes image defects (e.g. non-uniform illumination, electronic noise, glare effect), we use virtual slide scanner as the source of experimental subimages. To the best of our knowledge, there is no existing study in the literature addressing high-resolution, wholly-scanned histopathologic slide understanding in the subject of liver slides.

## Methods

### Experimental design and data preparation

In this section, we briefly describe histopathological slides we used in this research. We choose liver being source of the sample tissues because it is very likely targeted organ of metastatic tumors. We studied on 575 subimages extracted from two virtual slides of different specimens that are digitalized at the Department of Pathology, University of Arkansas for Medical Sciences. The virtual slides are visually and technically summarized in Additional File 1. Glass slides were scanned at 200× magnification using an Aperio ScanScope T2 scanner (Aperio, Vista, CA), and archived in 24-bit color JPEG 2000 format. The sizes of scanned slides vary and are up to 4 GB of uncompressed data, which reflects a region of 16 × 16 mm of a glass slide.

The slides represent two de-identified sections formalin-fixed, paraffin-embedded liver with metastatic colon carcinoma. Use of tissue was approved by the UAMS Institutional Review Board. Blocks were sectioned at 4 *μ*m and double-immunostained using anti-smooth muscle actin (SMA, Monoclonal Mouse Anti-Human Alpha Smooth Muscle Actin-7 ml-Code # N1584-Dako Corporation) and anti-CD31 (Monoclonal Mouse CD31, Endothelial Cell, Clone, (1A10), Ventana Medical Systems) antibodies with the Dako Envision Double-Stain System Kit according to manufacturer's directions. Smooth muscle actin labels pericytes, which are closely associated with maturing blood vessels, and CD31 labels endothelial cells.

The aim of manual examination is to detect region-of-angiogenesis if any exist. Hence, the main task of this study is to accurately identify those fixed-size-regions in which angiogenesis formation is seen. Note that in the subimage classification step of this study, a subimage is a positive subimage if it raises enough clinical interest for further investigation of angiogenesis; and a negative subimage if does not. At this preprocessing step, one of the challenges is choice of subimage size that is crucial for a reliable learning environment. The size of the subimage should be chosen with care regarding following statements:

- Selected subimage size should be resistant to orientation of vessel formation.

- Subimages should be consistent with its label when classified (i.e., a subimage should not be positive and negative at the same time).

- Subimages should be large enough to be judged by a classification algorithm.

Obviously, there is no fixed subimage size and shape for angiogenesis detection since magnification level and dimension of targeted components varies significantly with different tissue. A fixed subimage size 128 × 128 pixels is reported for head and neck carcinoma in [18], whereas dynamic ones were used in [19] for segmentation of squamous epithelium from cervical histological virtual slides. As for this study, an experiences pathologist (LH) evaluated many cases and reported 300 × 300 pixels (0.14 × 0.14 mm) being the best selection for this research. Based on this finding, the size of the subimages is fixed to 300 × 300 pixels through the framework. A subimage having this size was found to be both large enough to include angiogenesis of clinical interest but small enough to be label as positives or negatives.

**Figure 2:**
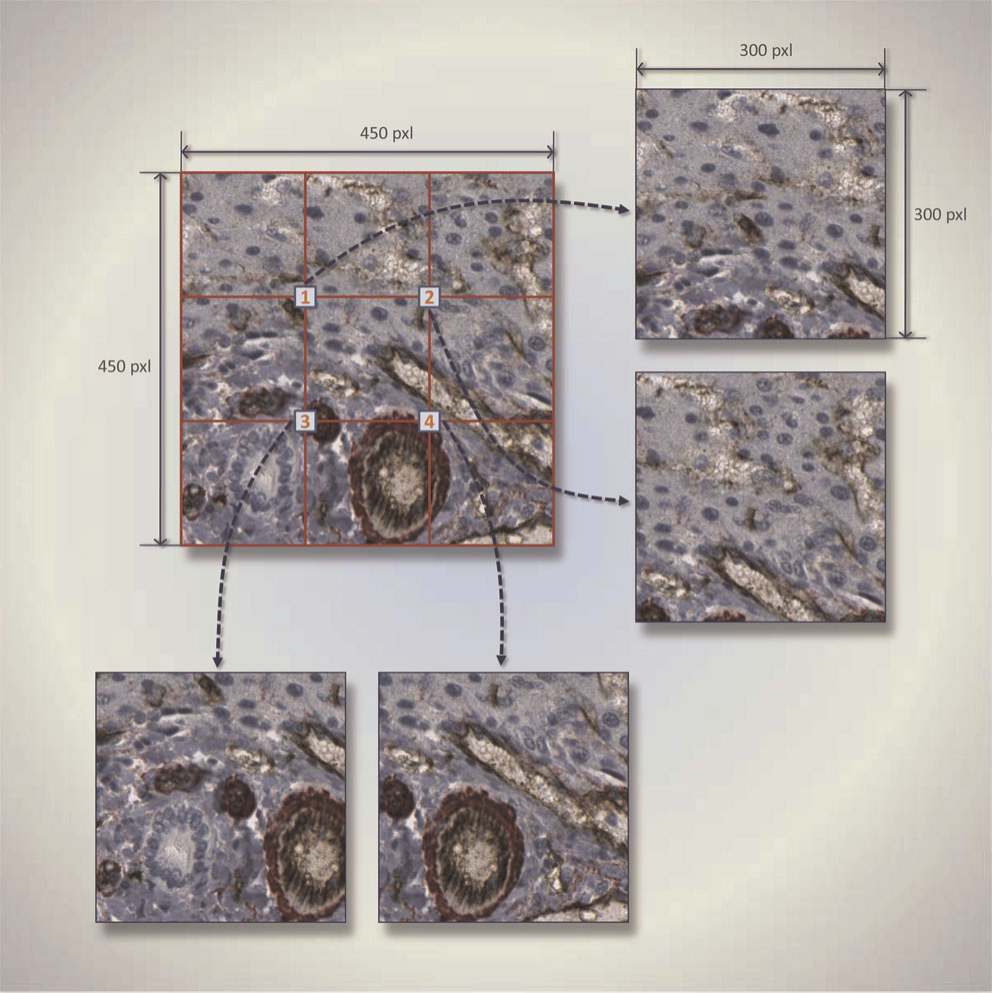
**A partial image layout for a 450 × 450 pixels image that is partitioned into four blocks**. This figure shows an example of how a 450 × 450 pixels image is partitioned into four 300 × 300 subimages. The four numbered squares are the centers of the subimages. Each subimage has a 50% overlap with neighboring subimage.

A general flowchart of this study is given in Additional File 2. To label angiogenesis regions, each virtual slide is partitioned in such a way that each subimage has a half overlap with the next subimage in four directions, if possible. Incomplete subimages on the last column or last row are ignored. Figure 2 depicts an example layout for a 450 × 450 pixels image, which is partitioned into four subimages. Since we guarantee each subimage is 50% overlapped by adjacent subimages, we approximately obtain

subimages for each virtual slide.

Training dataset is captured at random points in two slides. We first collect 214 positive and 204 negative subimages from slide S_1 _and 80 positive and 77 negative subimages from slide S_2_.

**Figure 3:**
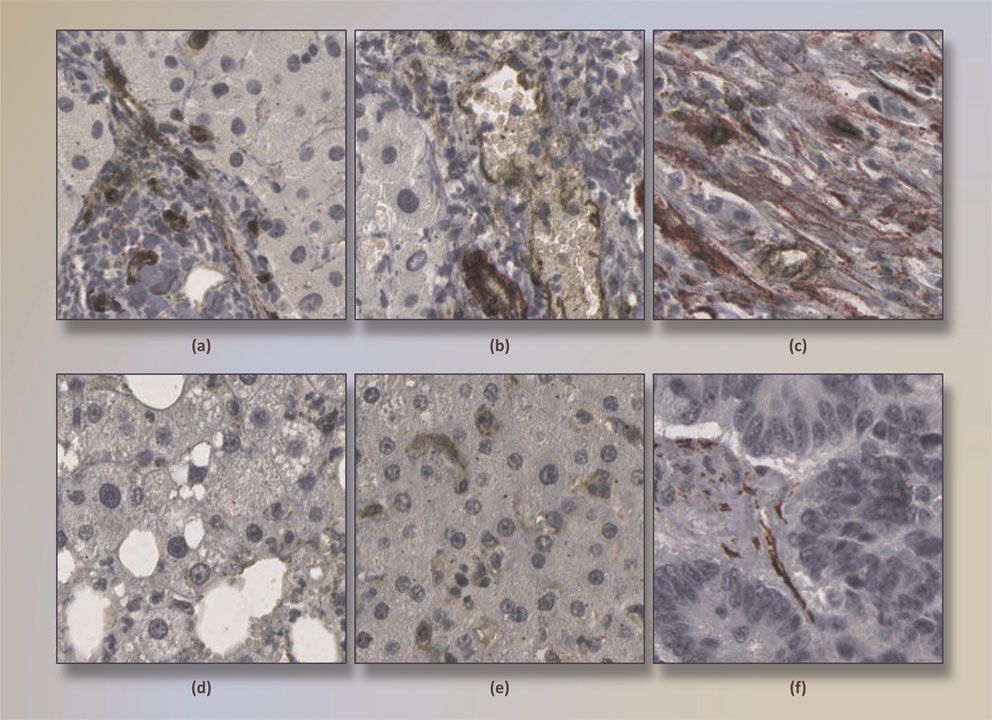
**Six training images**. Three positives and three negatives are given on the first and second row, respectively. In the images of first row, the association of red and brown regions is obvious. The image (c) has a few pericytes and many fibroblasts together. However, this image was selected as positive due to presence of targeted vessel. The image (d) has 903 red pixels that are spread out in the image. Therefore, there is not a perception of red region. Another negative image (f) 1888 red pixels in a few region, but they are not found with a brown stained regions.

To reduce inconsistency in training dataset, the pathologist labeled subimages twice with two-week gap between them. Subsequently, we are left with 248 positive and 274 negative subimages, i.e, 53 (46 positive and 7 negative) subimages having different labels at different assessments were removed from the training set. Note that the during selection of training subimages, it is crucial to choose subimages that present a wide range of positive and negative structures. Thus, care must be taken in the stage of training dataset construction; otherwise, misleading subimages would negatively affect the classification model. We avoid selecting fuzzy subimages and try to capture clear-to-classify subimages, because different regions of a virtual slide generally differ in density of cell, supportive tissue, lipid, and air. In Figure 3, we illustrate six training images, three positives and three negatives on the first and second row, respectively.

### Color classification

In the data preparation step, we apply neither histogram equalization nor filtering. Unlike some earlier studies [20,21], we opt to exploit every single pixel of the subimage. This is because even small area of a few reddish pixels can affect pathologist decision. Obviously color carries out a lot of information. Human perception indicates that roughly four groups of color are presented in the virtual slides used in this research: white, blue, brown, and red. Of these colors, we are interested especially in red and brown because brown staining labels endothelial cells, and red staining highlights pericytes. It is important to note that to correctly identify mature blood vessels, the red stained cells must be adjacent to brown endothelial cells. A red region not associated with brown-stained endothelium is more likely to indicate fibroblasts or smooth muscle.

To model four colors, we sampled each color from slides and train a decision tree classifier [22] in Red, Green, Blue (RGB) color space, which is the native color space of virtual histological slides.

*Definition 1*: Let *C*_*DT*_: *c *→ *y *∈ *{white, blue, brown, red} *be a decision tree classifier induced from sample colors; and map a color *c *of pixel to a class label *y*.

**Figure 4:**
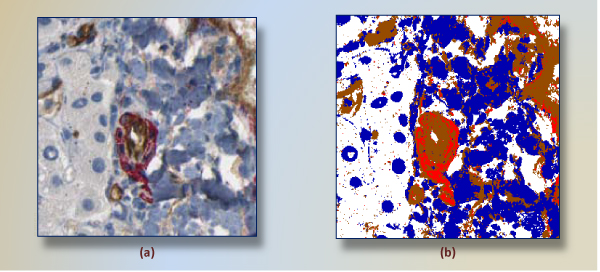
**A subimage segmented into four colors**. A positive subimage (a) and its four-color-segmented (white, blue, red, brown) corresponding (b) are shown. This subimage has a mature vessel around center. In (b), same region is segmented mainly two regions, red and brown.

The resulting classifier serves as pixel classifier in the framework. The reason to prefer the decision tree was its interpretability, where generated rules can be evaluated easily. The tree allows one to detect the set of rules and particularly the most important features in the decision-making. A widely used decision tree implementation of c4.5 [22] was run for color classification. Classification model *C*_*DT *_obtained from training dataset (191,238 samples from four groups) results in correct classification rate of 96%. We observe that less saturated (very bright) and low-light (dark) color points cause the most of the misclassification in the experiments. Figure 4 shows a positive subimage and its pixel-classified correspondings according to *C*_*DT*_.

### Preprocessing in pixel-classified subimages

Finding main colors in subimages, as seen in Figure 4, is essential to draw a virtual line between structures. However, spatial arrangements of pixels and size of a blob are obviously neglected during that process. Referring to the segmented image in Figure 4, there are several groups of discrete pixels in other regions, such as the scattering of red pixels in regions profusely occupied by browns. With the aim of separating each of structures from others in a more reasonable way, we cluster each group of pixels separately.

Cluster analysis is an important data mining task for finding meaningful groups in a dataset. In the literature, various clustering algorithms including density-based, graph-based, hierarchical, and K-means are applied to diverse applications such as web mining, pattern recognition, fraud detection, and meteorology [22].

**Figure 5:**
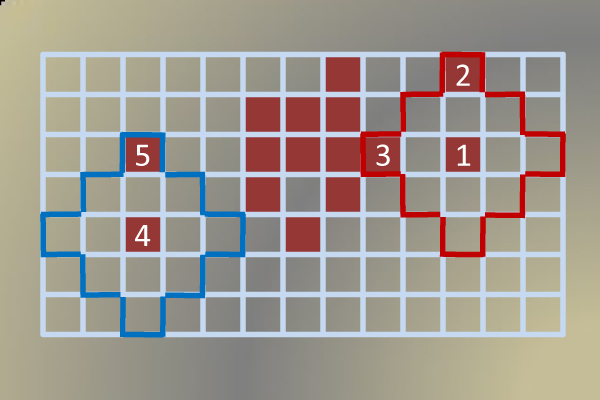
**A toy example depicting notion of DBSCAN clustering algorithm, with *ε *= 2 and MinPxl = 3**. Maroon pixels represent data points in search space (grid, or image). A neighborhood search fired at point 1 encloses the region drawn in red. This region has three points (1, 2, and 3) that is equal to or greater than MinPxl. Therefore, we expand the main body of cluster (a tight group of maroon points) to have points 1, 2, and 3. On the other hand, query for point 4, drawn in blue, has only two points that does not meet MinPxl. As a result, point 4 and 5 can neither form a new cluster nor merge an existing cluster. They will be reported to be outliers at the end.

**Figure 6:**
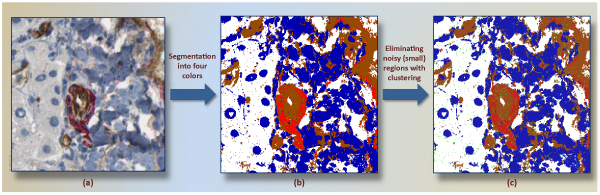
**Clustering steps**. A positive subimage (a), its segmented (b), and clustered with DBSCAN (c) corresponding. A close look at (c) reveals small green regions that are represent noisy groups of pixels of (b). For example, the endothelium region around the center of image has many small red regions in (b). However, they are too small to be counted as pericytes or other structures since they are so small and far from other same kind of structure. Hence, they are removed and only significant structures are kept in subimage (c).

From the standpoint of the angiogenesis detection, we redefine and make use of a density-based approach, namely DBSCAN (A Density-Based Algorithm for Discovering Clusters in Large Spatial Databases with Noise) [18,23]. DBSCAN is significantly more effective in discovering clusters of arbitrary shapes, which perfectly fit the task of detection of different structures. It was successfully used for synthetic dataset as well as earth science and protein dataset [23]. We omitted a theoretical background of DBSCAN and refer the reader to [23] for detailed explanations. In DBSCAN, pixels quite separated from main body of its source component are considered as outliers in the data. Therefore, DBSCAN in 2D was employed to find more isolated tissue component in subimages. The parameters *ε *and *MinPxl *are set to 2 and 3, found experimentally. With the use of euclidean distance, these two parameters mean that if we find at least three pixels in the two-pixel-neighborhood of a query pixel, this region will be merged to originating body of cluster. A toy example is given in Figure 5. In addition, two-step approach, in which a raw subimage is converted to clustered regions, is shown in Figure 6.

### Image processing and feature design

As a general term, feature design is to describe objects to represent them in more distinguishable manner for a learning system. Technically speaking, a feature obtained from an image is an output of certain function or algorithm. However, a single parameter representing the entire image is insufficient to describe targeted content of image in most cases. Thus, it is needed to characterize an image by extracting a set of features rather than a single one. Furthermore, it is widely accepted that feature design is one of the most significant steps in the area of machine learning, data mining, pattern recognition, and content-base image retrieval. Earlier studies [13,24] show that there exist several ways to present image data via different feature selection schemes.

In this section, we shed light on the set of features that will be used within classification schema.

#### 1. Region features

With an assumption that the amount of each group of pigments in each subimage is correlated with the decision of pathologist by some means; the number of pixels for each cluster is reported as area features: red P_A_, brown E_A_, white W_A_, and blue B_A_. Additionally, we extract another shape feature since elongated shape of fibroblasts is hallmarks of new formations. To quantify shape of red stained regions, we calculated *weighted average circularities of red regions*. First, circularity of each red region was found by

**Figure 7:**
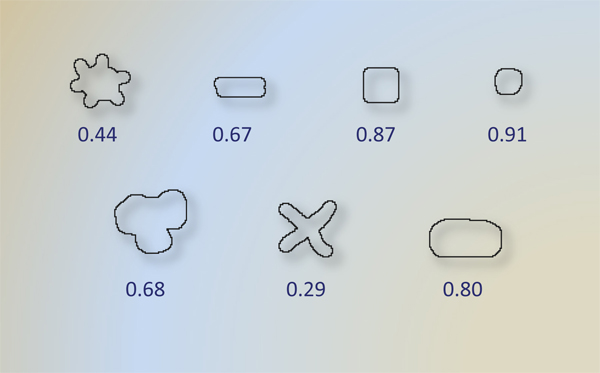
**Circularity of sample shapes**. To distinguish oblong stain fibroblasts from other red stained structures, we exploited this measure at the feature design step. The low circularity with low Γ *(P, E) *value points fibroblasts out in current subimage.

Figure 7 shows circularity values of a group of closed shapes. Note that circularity is calculated for boundary of connected regions, i.e., we calculated circularity for each separated red regions. Subsequently, we found area weighted average of circularity if more than one red stained regions are detected in the subimage. Area weighted average is given by

where CT_i _and A_i _are circularity and area of i^th ^red stained region, respectively. If only one region is found, area weighted average is equal to the area of the single region.

#### 2. Morphologic and spatial features

Being disorganized, excessively branched, tumor-induced angiogenesis show different morphology from healthy vessel network [25]. Many morphological features, such as shape, diameter, branching pattern, and tortuosity, are statically proven to be useful features of vessels within malignant tumors [26,27]. Also, a lumen surrounded by brown-stained endothelium suggests a mature capillary. In contrast, isolated endothelial staining without any associated red-stained pericytes indicates immature blood vessel. Therefore, we first suggest using region dilation, one of fundamental mathematical morphologic operations. This operator is commonly called *fill, expand, or grow*. Simply, effect of this operation on a binary image is expanding the boundary of a region using a structuring element. Direction of expansion can be inward if region has a hole in it.

Upon used with binary images, where each pixel is either 0 or 1, structuring element is first centered with a nonzero pixel of the image, then each pixel of the structuring element is added to underlying region of binary image using logical OR operator. Formally, given an image A and structuring element B, dilation of A by B is expressed by [28]

**Figure 8:**
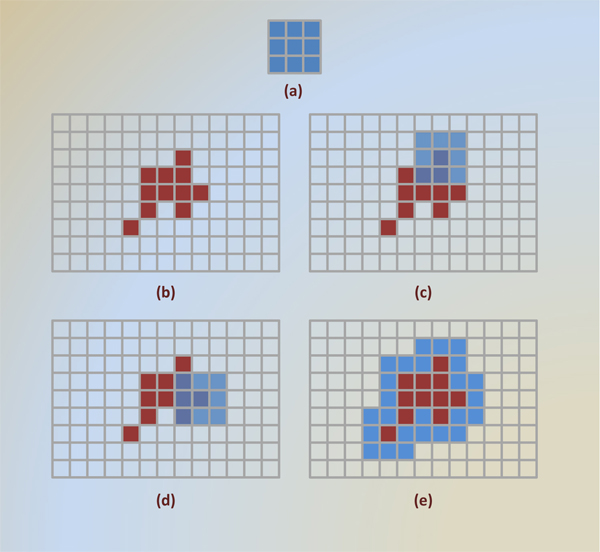
**A toy example showing dilation operation in binary images**. 3 × 3 pixels structural element is given (a). A group of 11 pixels (b) is the experimental image. In (c), structural element is centered with a pixel of base image, and six new pixels added to original image at this steps. In another step (d), structural element is centered with the most left pixel of image, and five surrounding pixels are converted 0 to 1. Final outcome is given in (e), where blue ones represent effect of dilation operation.

**Figure 9:**
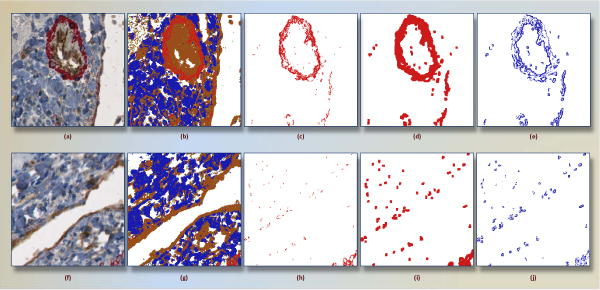
**Intermediate steps in computation of Γ (P, E) for positive (a) and negative (f) subimages**. Images (b) and (g) show clustered regions for their originals. Only red regions of (b) and (g) are give in (c) and (h), respectively. Morphological dilation operation expands (c) as seen in (d), and (h) as in (i). For the positive image, overlapping pixels of red regions of (d) and brown region of (b) is given in (e). In same manner, (j) represents overlapping pixel for negative subimage. The corresponding areas (9857 pixels) in (e) and (4896 pixels) in (j) clearly help classifier distinguish both subimages.

Figure 8 graphically depicts a binary input image with a few intermediate steps of dilation operation. To use within our classification framework, we calculate overlapping area of dilated-region  and non-dilated one *R*_*z*_. This measure quantifies how two regions spatially related. For instance, a dilated red stained region overlapping with large portion of endothelium implies that i) both regions are spatially close, ii) red stained region are high likely composed of pericytes, and iii) red region is big enough to be noticed by a pathologist. Unlike this situation, small area of intersecting region means that red stained regions and endothelium formations are independent from each another; and points fibrosis out with the presence of large red stained regions. Figure 9 explains mentioned observation with two expressive examples. The notation R^D ^indicates result of dilation operation of *R *with a structural element *B *that is 5 × 5 square block of pixels in this study.

Based on dilation operation detailed above, we define intersection function as follows:

Verbally this function returns pixel area of intersecting regions of dilated R_1 _and non-dilated R_2_. We build features from intersection of i) dilated red stained regions with endothelium Γ (P, E), ii) dilated endothelium with red stained regions Γ (E, P), and iii), dilated endothelium with white regions Γ (E, W). The second feature Γ (E, P) is intended to quantify possible pericytes around endothelium. Third one is designed to find lumen region around endothelium.

Raw features obtained from dilation operations are numeric, which are measurements of areas as seen in Figure 9(e) and 9(j). In addition to pixel counts, the ratio of intersecting region(s) to some other related region(s) is reported as extra features. For the interaction of red stained regions and endothelium, this relation is defined as follows: Γ (P, E)/P, where both, nominator and denominator are area measures. Similarly, Γ (P, E)/E is used as one of secondary morphological characteristics.

#### 3. Fractal features

The term *fractal*, meaning broken, was coined by Benoit Mandelbrot in 1975. Fractals rose from dealing with the datasets with completely natural or irregular structures [29]. Technically fractal measures are applied to irregular or fragmented sets that show the property of self similarity at different levels of magnification [30]. Applications in image compression [31] and image coding [32], object modeling, representation and classification [33] benefit from fractal analysis significantly. Recently, Luzi et al. [34] reported that fractal analysis has a good index that distinguishes between tumors of different histological types, both low and high grade, in the urothelial neoplasia. Thus, to understand the complexity of red stained region and endothelium in a subimage, we compute fractal dimensions for each 300 × 300 pixels subregion.

One of the most common methods is the estimation of the box dimension [35]. Given a bounded set A in 2D euclidean space, consider laying out a grid paper over the image, where the side of each box is size h. We then find number of nonempty boxes, given by N(h). In this method, fractal dimension is given by

**Figure 10:**
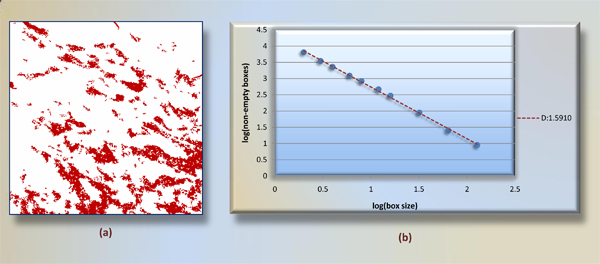
**Fractal calculation with box-counting method**. A binary subimage is given (a), where red pixels are kept only. Number of nonempty boxes in a grid setting with box sizes 2, 3, 4, 6, 8, 12, 16, 32, 64, and 128 are found first. Then negative slope induced from these values in log-log plot (b) is reported as fractal dimension of (a).

Since smaller boxes give more accurate estimation of N(h) and different magnification do not give same ratio in complex shapes, a log-log plot (resolution scale vs. number of windows occupied) is used to determine fractal dimension. Fractal dimension D is the slope m of the line of best fit, D = m. These features are denoted as D(P) and D(E), respectively. Note that in contrast to region features, we consider red stained regions as a set of pixels, not individual areas. Thus, the box-counting method is more appropriate to use herein. A sample subimage and its log-log plot are given in Figure 10.

In summary, for each subimage we obtain five region-based, five morphological and two fractal dimension features, which are shown in Table 1.

**Table 1:** Ranking of proposed features with information gain

Rank #	Feature	Type	Info. Gain
1	*D(P)*	Fractal	0.4555
2	Γ*(E, P)*	Morphological	0.4401
3	*Circ(P)*	Region	0.4301
4	Γ*(P, E)*	Morphological	0.3555
5	*W*_*A*_	Region	0.293
6	Γ*(P, E)/E*	Morphological	0.287
7	*P*_*A*_	Region	0.2643
8	Γ*(E, W)*	Morphological	0.2637
9	*E*_*A*_	Region	0.1191
10	Γ*(P, E)/P*	Morphological	0.1143
11	*B*_*A*_	Region	0.0808
12	*D(E)*	Fractal	0.0587

### Classification of subimages

As a trendy statistical classification model, the Support Vector Machines (SVMs) have been extensively applied to numerous classification problems in the last decades. We preferred this algorithm for its robustness and good performance to tackle two-class classification problem. From the SVMs' viewpoint, classification problems are grouped into i) linearly separable and ii) linearly non-separable cases. In terms of solution, formulation of a non-separable problem also comprises the separable one. Furthermore, real-world problems are most likely linearly non-separable. Therefore we herein present SVMs formulation to make the solution more comprehensive and handle the cases with imperfect separation.

Consider the problem of separating a set of training dataset S, denoted by

where |*S*| = *l *is the size of training set. In two-class classification problems, we try to infer a function

Support vector machines intend to find the optimal separating hyperplane that maximizes the margins from positive and negative samples in feature space. The classification function has given in the form

where w is normal to hyperplane and b is a bias term (not to be confused with statistical bias), which should meet the following condition:

As the measurement of violation of the constraints [16], nonnegative slack variables *ξ*_*i *_are introduced to relax non-separable cases. Then, optimal hyperplane is the one minimizes the following summation:

where C is a parameter used to penalize variables *ξ*_*i*_.

For a linearly non-separable case, there is one more technique to increase accuracy of the system: kernel trick. With this, training vectors x_*i *_are mapped from original feature space H into a high dimensional feature space H by a non-linear transformation Φ (x). The training vectors are expected to become linearly separable in the new feature space. Then the optimal hyperplane in H is sought as described before. Since solving the system only involves in inner products (x_i_, x_j_) in H, a kernel function k = (x_i_, x_j_) is used to solve the problem in H. Validity of kernel function is ruled by Mercel's theorem [36]. Basically speaking, any kernel whose gram matrix is symmetric positive definite is regarded as a valid kernel in an inner product space. Polynomial kernel (x_i_, x_j_)^d ^and radial basis function kernel  are of widely used ones in the literature.

### Statistical analysis

In experiments, primary measures, recall (sensitivity), precision, and f-measure, are used to judge classification results. These measures rely on true positive (TP), false positive (FP), true negative (TN), and false negative (FN) numbers of classification. To avoid confusion with the terms used to evaluate our system, we refer reader to the confusion matrix given in Table 2. Recall is known as the percentage of positive labeled instances that were predicted as positive and found by . Precision is defined as the percentage of positive predictions that are correct, and calculated as . An alternative to success rate ((TP+TN)/(TP+FP+TN+FN)), f-measure is the harmonic mean of the precision and recall, and calculated by . Within current definition of f-measure, we give equal weights to recall and precision, whereas some studies adopt various weighing pairs depending on important of target measures.

**Table 2:** Confusion matrix for binary classification used in this study

		***Actual***	
		
		Positive	Negative
		
***Test***	Positive	TP	FP
	Negative	FN	TN

## Results and discussion

Since microvessel formations play a key role in metastasis evaluation and prognosis of base tumor, in this study we proposed an automatic framework to quantify mature microvessels in histopathological images of liver carcinoma tissues.

### Feature ranking

Before the details of results section, we will take a closer look at extracted features. Since the classification power of an extracted feature is unknown and subjective at this point, we measure discriminating ability of each feature over groundtruth subimages. Information gain that indicates the amount of information a feature has in the system, is used to rank the features. A feature with greater information gain is more informative than those with less information gain. We employed information gain [37] to rank 12 features. The ranking of proposed features is given in Table 1. Accordingly, fractal dimension of red stained regions D(P) is the most helpful feature in the classification of subimages, whereas, interesting enough, fractal dimension of endothelium D(E) is the most weakest one. Furthermore, Γ (E, P) and Circ(P) have fairly close values to D(P), which shows that both are good features like D(P).

Selecting a subset of relevant features from a given set (in our case, 12 features) can be increase accuracy of classification; however, we carried out experiments using all of 12 features because finding the most informative subset from a group of features is out of scope of this communication.

### Experimental results

Twelve features were calculated for each training subimage, and labeled either positive or negative under pathologist's directions. Structural element used to dilate corresponding red or brown stained regions is 5 × 5 pixels filled square, whereas 3 × 3 used in the toy example of Figure 8. In the calculation of fractal dimension, we used box sizes 2, 3, 4, 6, 8, 12, 16, 32, 64, and 128 to find *D *of red stained regions and endothelium in each subimage.

At the classification phase, we normalized all features in the range of [-1, +1]. We conducted extensive experiments on our subimage database with many combinations of different parameters (*degree: 1, 2,..., 6; γ: 2*^-1^, 2^0^,..., 2^-6^; *C*: 2^-1^, 2^0^,..., 2^12^) and kernels. It is reported in [38] that for best replicability scores, single run of randomized 10-fold cross validation is not dependable enough for unbiased comparison of outcomes. For this reason, we did 10-time randomized 10-fold cross validation for each parameter set.

The radial basis function (RBF) kernel gives better overall f-measure when compared to others, which are linear, polynomial, and sigmoid kernels. Hence, we only report results of RBF kernel, where element of kernel matrix is expressed by . One of two primary parameters of RBF kernel *γ *takes place in kernel function. Another one *C *is that the penalty parameter of the error term. In experiments with the RBF kernel, we vary *γ *iteratively in [2^-1^, 2^-2^,..., 2^-6^] and *C *in [2^-1^, 2^0^,..., 2^12^]. Table 3 and Table 4 summarize grid parameter search for *γ *and *C *regarding recall-precision pair and f-measure, respectively. In Table 3, the overall highest recall-precision pair (0.9113, 0.8958) is obtained with C = 2^11 ^and *γ *= 2^-5^. Although we obtained very close values, in both tables the highest values are shown in bold. Additionally, we tested significance of f-measures over 10-time randomized 10-fold cross validation results, which have normal distribution, not skewed. T-test is experimented to identify if accuracy differs significantly. Nadeau and Bengio [39] pointed out that independence of sample set is important to avoid test set overlap and can be worked out by considering the variance estimate. This process is called corrected resampled t-test. In Table 4, we compared distribution of the highest f-measure (C = 2^11 ^and *γ *= 2^-5^) with others. Corrected resampled t-test with a 5% significance level was used to test null hypothesis H_0_: *μ*_1 _= *μ*_2 _against H_a_: *μ*_1 _<*μ*_2 _and H_a_: *μ*_1 _>*μ*_2 _for results of each pair of different parameters. In summary, for the underlined cells, such as C = 2^1^, *γ *= 2^-2 ^and C= 2^3^, *γ *= 2^-2^, we cannot reject null hypothesis H_0 _in favor of alternative hypotheses, which state that means of two distributions do not differ.

**Table 3:** Recall – Precision obtained from grid search with parameters *γ *and C using RBF kernel.

		**γ**					
		
		**2**^-1^	**2**^-2^	**2**^-3^	**2**^-4^	**2**^-5^	**2**^-6^
		
***C***	**2**^1^	0.91 – 0.85	0.92 – 0.85	0.92 – 0.84	0.92 – 0.83	0.92 – 0.82	0.9 – 0.82
	**2**^2^	0.9 – 0.86	0.91 – 0.85	0.91 – 0.85	0.91 – 0.84	0.91 – 0.83	0.91 – 0.82
	**2**^3^	0.91 – 0.87	0.9 – 0.86	0.91 – 0.86	0.91 – 0.85	0.91 – 0.84	0.91 – 0.83
	**2**^4^	0.91 – 0.88	0.91 – 0.87	0.91 – 0.86	0.91 – 0.86	0.91 – 0.85	0.91 – 0.84
	**2**^5^	0.92 – 0.89	0.92 – 0.89	0.92 – 0.87	0.91 – 0.86	0.92 – 0.86	0.91 – 0.85
	**2**^6^	0.91 – 0.89	0.92 – 0.89	0.92 – 0.89	0.92 – 0.87	0.91 – 0.86	0.91 – 0.86
	**2**^7^	0.89 – 0.89	0.92 – 0.9	0.91 – 0.89	0.93 – 0.88	0.91 – 0.87	0.91 – 0.87
	**2**^8^	0.89 – 0.89	0.91 – 0.9	0.91 – 0.89	0.92 – 0.89	0.92 – 0.87	0.91 – 0.87
	**2**^9^	0.88 – 0.89	0.88 – 0.89	0.91 – 0.9	0.92 – 0.9	0.92 – 0.89	0.91 – 0.87
	**2**^10^	0.88 – 0.88	0.87 – 0.89	0.9 – 0.9	0.91 – 0.89	0.92 – 0.9	0.92 – 0.88
	**2**^11^	0.87 – 0.87	0.86 – 0.88	0.89 – 0.89	0.91 – 0.89	**0.92 – 0.9**	0.92 – 0.89
	**2**^12^	0.87 – 0.88	0.86 – 0.88	0.87 – 0.89	0.91 – 0.9	0.91 – 0.89	0.92 – 0.9

**Table 4:** F-measures of grid search with parameters *γ *and C using RBF kernel

		**γ**					
		
		**2**^-1^	**2**^-2^	**2**^-3^	**2**^-4^	**2**^-5^	**2**^-6^
		
***C***	**2**^1^	0.8740	*0.8762*	0.8729	0.8630	0.8583	0.8524
	**2**^2^	0.8736	0.8757	0.8750	0.8686	0.8603	0.8560
	**2**^3^	0.8843	*0.8742*	0.8759	0.8743	0.8656	0.8594
	**2**^4^	*0.8899*	*0.8849*	0.8790	0.8784	0.8721	0.8663
	**2**^5^	*0.8951*	*0.8960*	*0.8859*	*0.8792*	*0.8798*	0.8708
	**2**^6^	*0.8926*	*0.8966*	*0.8971*	*0.8854*	*0.8807*	*0.8784*
	**2**^7^	*0.8853*	*0.9008*	*0.8956*	*0.8970*	*0.8806*	*0.8810*
	**2**^8^	*0.8808*	*0.8965*	*0.8945*	*0.9009*	*0.8898*	*0.8826*
	**2**^9^	*0.8790*	*0.8802*	*0.8977*	*0.8999*	*0.8972*	*0.8868*
	**2**^10^	0.8715	*0.8741*	*0.8939*	*0.8945*	*0.9016*	*0.8935*
	**2**^11^	0.8659	0.8669	*0.8808*	*0.8940*	**0.9018**	0.8970
	**2**^12^	0.8641	0.8640	0.8713	*0.8953*	*0.8945*	*0.9002*

Results in underlined cells do not significantly differ than results of C = 2^11^, *γ *= 2^-5^.

## Discussion

Since an image itself is a structured data in 2D, feature extraction approaches might be considered as dimension reduction tools that can be employed at different levels of complexity. In the literature of computer vision, types of features can be roughly categorized into i) global features and ii) local (regional) features. In this framework, an individual subimage would be an input object, because the goal is classification of subimages. Therefore, all features are global since we do not partition 300 × 300 pixels subimages into smaller ones.

The number of subimages we used was influenced by three factors. First, we wished to estimate agreement with a high level of precision. Second, we wished to precisely measure estimates of sensitivity and specificity. To do this, we decided that approximately half the subimages should represent regions of active angiogenesis and half regions where angiogenesis is not evident. Finally, we had to consider practical limitations regarding the time the pathologist had to devote to the project. After consulting with the pathologist, we decided that the study would need at least 500 confirmed subimages. With this number, the estimate of the standard error of agreement would be no larger than 2.2%, while the standard error of the sensitivity (specificity) would be no larger than 3.2%. To obtain this sample, we acquired approximately 600 subimages to be reviewed by the pathologist. From these subimages, 248 were confirmed to represent regions of angiogenesis and 274 were confirmed to represent regions of no angiogenesis.

Illumination of the histological slides and tissue color pigments change slightly due to some natural factors, laboratory conditions and human intervention to the staining process. The variable resulting from operator can be corrected with use of fully-automated sectioning devices. However, other inputs, such as i) temperature of tissue, staining chemicals or antibodies, and room; ii) procedural differences in staining techniques; iii) lighting condition during digitization, are significantly affect color variation in digital images. These mentioned factors are out of scope in this study, and we assume that all virtual slides are obtained under same conditions. Therefore, the use of virtual slides is highly preferable to other regular digital camera because virtual slide scanners eliminate operator involvement and takes all snapshot of whole slide in the same lighting conditions. Note that to deal with color invariance in virtual slides, we need to only repeat color classification section of whole framework. This is because the outcomes of all image features given in methodology section differ considerable with variation of color labels fed by color classification step.

**Figure 11:**
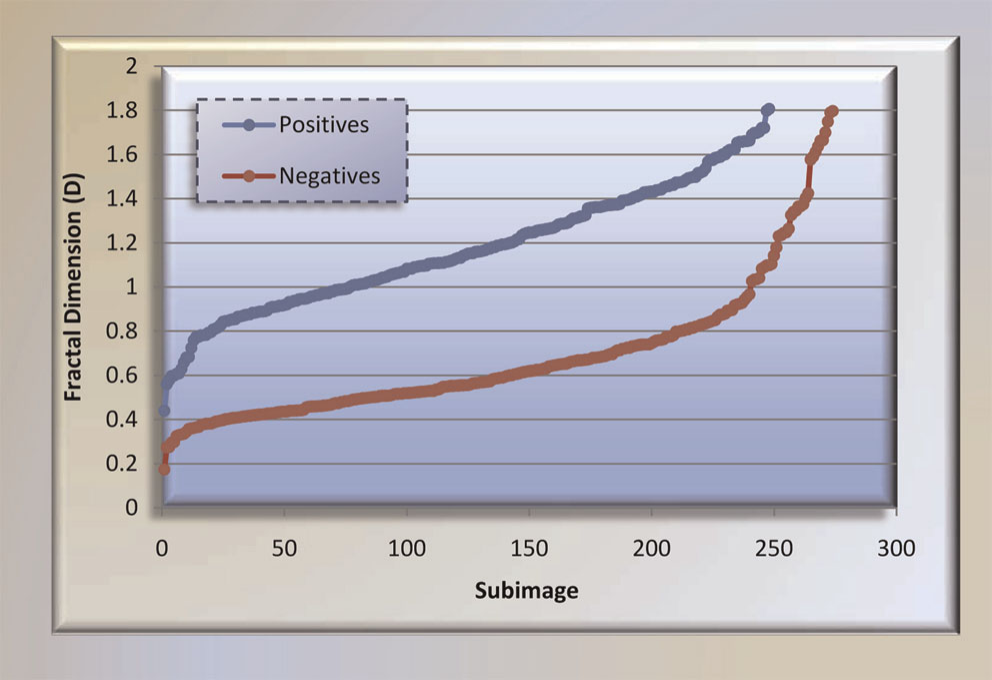
**Fractal dimensions of positive and negative subimages**. We show ordered fractal dimensions of positive and negative subimages. The reason is that this feature is ranked first as most distinguishing feature in all 12 ones within information gain analysis. For a reasonable threshold, for instance 0.8, 228 of 248 positive subimages have greater, 211 of 274 negative subimages have less fractal dimension than 0.8.

Being one of features fractal dimension shows a complexity of pixel's spatially. Looking at Table 1, D(P) is reported as the most helpful one in among all features; in contrast, D(E) ranks last. The outcome suggests that complexity of red stained pixels in a subimage is highly correlated with presence of angiogenesis therein. We depict D(P) for each class of subimages in Figure 11. Box counting method we used is fully resistant to 90° image orientations because of square size of subimages. However, other orientation angles would vary fractal dimension slightly.

**Figure 12:**
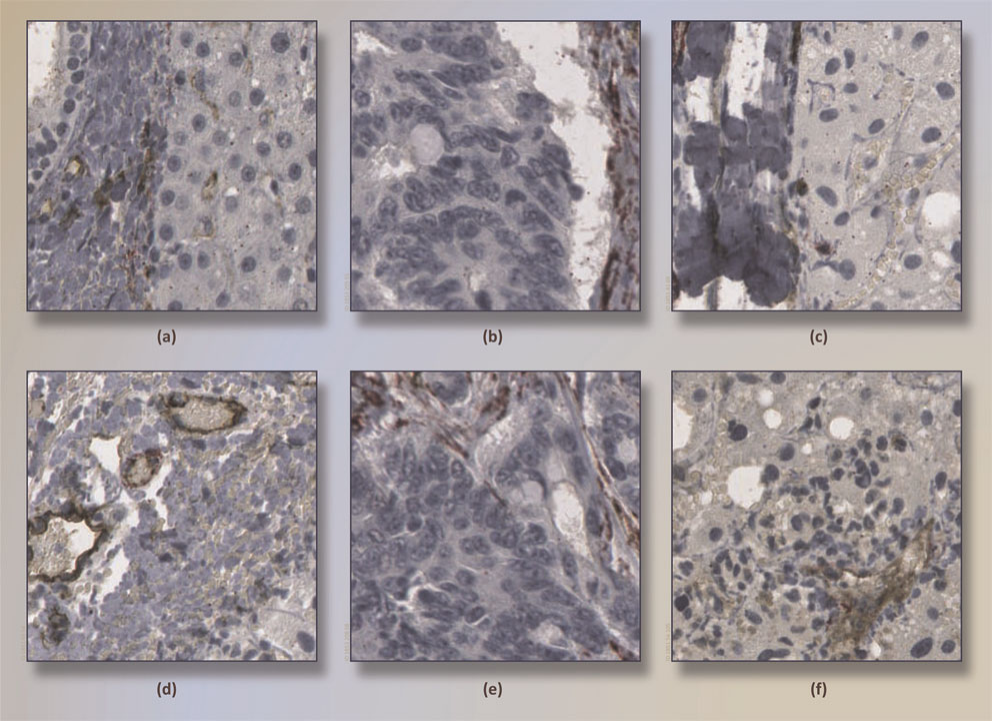
**Subimages that are classified incorrectly**. According to classification results (RBF kernel, C = 2^11^, *γ *= 2^-5^), false positives (a)-(c) and false negatives (d)-(f) are shown. Regarding (a), Γ (P, E)/P ratio and Γ (E, W) are high regarding other negatives in training dataset. Therefore, (a) is classified as negative, which actually is positive. The subimage (f) has angiogenesis because of a small pericytes regions is surrounded by endothelium around lower-left corner of image. Mistakenly, this image was classified as negative.

In general, f-measure of 0.90 is quite promising success for a pioneering framework that addresses angiogenesis identification in whole-scan liver tissues. With these results, false-positive ratio and false-negative ratio are respectively 0.08 and 0.09. Figure 12(a)–(c) and Figure 12(d)–(f) show examples of false-positives and false-negatives, respectively. For the image Figure 12(a), for instance, Γ (P, E)/P and Γ (E, W) are comparatively higher than other negative subimages. Therefore, this subimage, classified as negative by the pathologist, is labeled positive by framework. As another classification error, Figure 12(f) has angiogenesis because of a small pericytes regions is surrounded by endothelium around lower-left corner of image. Mistakenly, this image was classified as negative, i.e., amount of red stained region and its dilated intersection with endothelium were not enough to classify it as positive.

## Conclusion and research directions

In this study, we introduced a new classification framework in an effort to detect angiogenesis by examining virtual histological slides. We exploited notion of DBSCAN to create the most representing features in subimages.

The angiogenesis regions were investigated in equi-size subimages, which are obtained from the systematic partitioning of slides. Classification of subimages is achieved by a powerful classification algorithm, SVMs. We reported optimum recall, precision, and f-measure as the evaluation criteria. The experimental results validate our framework by providing average f-measure of 0.90, which means that framework missed a few positive subimages out and had reasonable false alarm rate on negative subimages.

Future work should be more on vicinity analysis between angiogenesis and negative subimages. This is, an ideal automated system should be take prior probability of subimages into consideration. Right now a subimage has an equal chance of being classified as positive or negative. This means that a known positive subimage does not affect the classification outcome of its neighboring subimages. Moreover, the current framework can be applied not only to angiogenesis detection in liver tissue but also to other cancers, such as breast and colon. The framework efficiency for various types of tissue will be explored in the future.

Currently, we only calculate set-based fraction dimension of components. Also some other techniques are available for region-based fractal calculation. These new methods will be considered for the future feature design tasks. At this time, only size of structure is taken into account as one distinguishing characteristics of subimages. However, two subimages having same size red stained region but different number of unconnected regions should not be treated as same. As another research direction, we will analyze each region (pericytes, endothelium, cell-cytoplasm-supportive material, and lumen, air, fat) in terms of size and distribution of sub-clusters.

## Competing interests

The authors declare that they have no competing interests.

## List of abbreviations used

VEGF: Vascular Endothelial Growth Factor; MVD: Microvessel density; RGB: Red, Green, Blue; CAD: Computer Aided Diagnosis; *μ*m: Micron, one millionth of a meter; C_DT_: Color Classification model employing Decision Tree algorithm; DBSCAN: Density-Based Clustering Algorithm; *ε*: Parameter of DBSCAN, defines size of a neighbor query; MinPxl: Parameter of DBSCAN, defines minimum number of point in a neighborhood query; P: Red region of a subimage; E: Brown region of a subimage; W: White regions of a subimage; B: Blue regions of a subimage; P_A_: Area of red region; W_A_: Area of white region; E_A_: Area of brown region; B_A_: Area of blue region; Circ(R): Area-weighted circularity of a given region R; ⊕: Dilation operator; Γ (A, B): Intersection operator for the regions A and B; N(h): Number of non-empty boxes given box dimension h; D(R): Fractal dimension of given region R; H: Feature space; k(): Kernel function; SVMs: Support Vector Machines; RBF: Radial Basis Function kernel for SVM; *γ*: Parameter of RBF kernel; C: Penalty parameter of the error in SVMs algorithm; TP: True positives; FP: False positives; TN: True negatives; FN: False negatives

## Authors' contributions

MM and UT have conceived the study. All of the authors participated in the overall design of the study. LH interpreted clustering results found by mention approaches. MM designed the algorithms, and features. MM developed the software and performed data analysis, algorithm testing, and benchmarking. HJS did sample size analysis. All authors contributed to the writing of this manuscript. All authors read and approved the final manuscript.

## Supplementary Material

Additional file 1 **Virtual Slides**. Two virtual slides we used in this study and their technical properties are summarized. Click here for file

Additional file 2 **General Flowchart of the Study**. Flowchart of this study is shown. Click here for file

## References

[B1] Jain RK Ward-Hartley KA Dynamics of cancer cell interaction with microvasculature and interstitium Biorheology 1987 24 117 125 3651585 10.3233/bir-1987-24205

[B2] Saeki T Tanada M Takashima S Takashima SH Saeki H Takiyama W Nishimoto N Moriwaki S Correlation between expression of platelet-derived endothelial cell growth factor (thymidine phosphorylase) and microvessel density in early-stage human colon carcinomas Jpn J Clin Oncol 1997 27 227 230 10.1093/jjco/27.4.227 9379508

[B3] Tsuji T Sasaki Y Tanaka M Hanabata N Hada R Munakata A Microvessel morphology and vascular endothelial growth factor expression in human colonic carcinoma with or without metastasis Lab Invest 2002 82 555 62 10.1038/labinvest.3780450 12003996

[B4] Belien A Somi S de Jong JS van Diest PJ Baak JP Fully automated microvessel counting and hot spot selection by image processing of whole tumour sections in invasive breast cancer J Clin Pathol 1999 52 184 192 10.1136/jcp.52.3.184 10450177 PMC501077

[B5] Slaton JW Inoue K Perrotte P El-Naggar AK Swanson DA Fidler IJ Dinney CP Expression levels of genes that regulate metastasis and angiogenesis correlate with advanced pathological stage of renal cell carcinoma Am J Pathol 2001 158 735 743 11159211 10.1016/S0002-9440(10)64016-3 PMC1850319

[B6] Sardari Nia P Stessels F Pezzella F Vermeulen PB Van Marck EA Van Schil P Growth index is independent of microvessel density in non-small cell lung carcinomas Human Pathol 2003 34 959 960 10.1016/s0046-8177(03)00247-8 14562297

[B7] Sarbia M Bittinger F Porschen R Dutkowski P Willers R Gabbert HE Tumor vascularization and prognosis in squamous cell carcinomas of the esophagus Anticancer Res 1996 16 2117 2221 8712753

[B8] Kawauchi S Fukuda T Tsuneyoshi M Angiogenesis does not correlate with prognosis or expression of vascular endothelial growth factor in synovial sarcomas Oncol Rep 1999 6 959 964 10425286 10.3892/or.6.5.959

[B9] Vaquero J Zurita M Coca S Salas C Oya S Expression of vascular endothelial growth factor in cerebellar hemangioblastomas does not correlate with tumor angiogenesis Cancer Lett 1998 132 213 217 10.1016/S0304-3835(98)00210-9 10397476

[B10] De Jong JS Van Diest PJ Baak JPA Heterogeneity and reproducibility of microvessel counts in breast cancer Lab Invest 1995 73 922 6 8558855

[B11] Belien JA Somi S de Jong JS Fully automated microvessel counting and hot spot selection by image processing of whole-tumour sections in invasive breast cancer J Clin Pathol 1999 52 184 192 10.1136/jcp.52.3.184 10450177 PMC501077

[B12] Erovic BM Neuchrist C Berger U El-Rabadi K Burian M Quantitation of microvessel density in squamous cell carcinoma of the head and neck by computer-aided image analysis Wien Klin Wochenschr 2005 117 1–2 53 7 10.1007/s00508-004-0298-3 15986592

[B13] Wester K Ranefall P Bengtsson E Busch C Malmström PU Automatic Quantification of Microvessel Density in Urinary Bladder Carcinoma British Journal of Cancer 1999 81 1363 1370 10.1038/sj.bjc.6693399 10604734 PMC2362966

[B14] Heikki L Jaakko L Tero V Pekka K Reidar G Morphometric analysis using automated image analysis of CD34-positive vessels in salivary gland acinic cell carcinoma Acta Oto-Laryngologica 2007 127 869 873 10.1080/00016480601053099 17763000

[B15] Bergers G Song S The role of pericytes in blood-vessel formation and maintenance Neuro Oncol 2005 7 4 452 464 10.1215/S1152851705000232 16212810 PMC1871727

[B16] Vapnik V 1995 The Nature of Statistical Learning Theory, Springer, NY8555380

[B17] Catalyurek UV Beynon M Chang C Kurc TM Sussman A Saltz JH The Virtual Microscope IEEE Transactions on Information Technology in Biomedicine 2003 7 4 230 248 10.1109/TITB.2004.823952 15000350

[B18] Mete M Xu X Fan CY Shafirstein G Automatic delineation of malignancy in histopathological head and neck slides BMC Bioinformatics 2007 8 Suppl 7 S17 10.1186/1471-2105-8-S7-S17 18047716 PMC2099485

[B19] Wang Y Turner R Crookes D Diamond J Hamilton P Investigation of Methodologies for the Segmentation of Squamous Epithelium from Cervical Histological Virtual Slides Proceedings of Machine Vision and Image Processing Conference 2007 83 90 full_text

[B20] Yagi Y Gilbertson J Whole Slide Imaging in Telepathology Third APT Telemedicine Workshop, 27–28 January, 2005

[B21] Tabesh A Kumar VP Verbel D Kotsianti A Teverovskiy M Saidi O Automated Prostate Cancer Diagnosis and Gleason Grading of Tissue Microarrays Proceedings of SPIE Int Symp on Medical Imaging, San Diego, CA 2005 5747 10.1109/TMI.2007.898536 17948727

[B22] Tan P Steinbach M Kumar V Introduction to Data Mining 2005 Addison-Wesley

[B23] Ester M Kriegel H-P Sander J Xu X A Density-based Algorithm for Discovering Clusters in Large Spatial Databases with Noise Proceedings of 2nd Int Conf on Knowledge Discovery and Data Mining. Portland, OR 1996 226 231

[B24] Dollar P Tu Z Tao H Belongie S Feature mining for image classification. In Computer Vision and Pattern Recognition In the Proceedings of IEEE Computer Vision and Pattern Recognition 2007 2007 1 8

[B25] Chang RF Huang SF Moon WK Lee YH Chen DR Computer algorithm for analysing breast tumor angiogenesis using 3-D power Doppler ultrasound Ultrasound in Medicine and Biology 2006 32 10 1499 1508 10.1016/j.ultrasmedbio.2006.05.029 17045870

[B26] Chang RF Huang SF Lee YH Chen DR Moon WK Breast tumor angiogenesis analysis using 3D power Doppler ultrasound Proc Proceedings of SPIE 2006 10.1016/j.ultrasmedbio.2006.05.029 17045870

[B27] MacDonald IC Chambers AF Intravital Videomicroscopy in Angiogenesis Research Methods in Enzymology 2008 444 Academic Press 201 230 19007666 10.1016/S0076-6879(08)02809-7

[B28] Dougherty ER Lotufo RA Hands-on morphological image processing 2003 SPIE Press, Bellingham, Wash, USA

[B29] Mandelbrot BB The fractal geometry of nature 1982 W.H. Freeman and Company

[B30] Falconer K Fractal Geometry: Mathematical foundations and applications 2003 West Sussex: John Wiley & Sons, Ltd

[B31] Cardinal J Fast fractal compression of grayscale images Image Processing 2001 10 1 159 164 10.1109/83.892452 18249606

[B32] Lee CK Lee WK Fast fractal image block coding based on local variances Image Processing 1998 7 6 888 891 10.1109/83.679437 18276301

[B33] Abate AF Distasi R Nappi M Riccio D Face authentication using speed fractal technique Image and Vision Computing 2006 24 9 977 986 10.1016/j.imavis.2006.02.023

[B34] Luzi P Bianciardi G Miracco C Santi MM Vecchio MT Alia L Tosi P Fractal Analysis in Human Pathology Annals of the New York Academy of Sciences 2006 879 1 255 257 10.1111/j.1749-6632.1999.tb10428.x 10415836

[B35] Keller JM Chen S Crownover RM Texture description and segmentation through fractal geometry Computer Vision, Graphics, and Image Processing 1989 45 150 166 10.1016/0734-189X(89)90130-8

[B36] Scholkopf B Smola A Learning with Kernels. Support Vector Machines, Regularization, Optimization and Beyond 2002 Cambridge, MIT Press

[B37] Mitchell TM Machine Learning 1997 The Mc-Graw-Hill Companies, Inc

[B38] Bouckaert RR Frank E Evaluating the replicability of significance tests for comparing learning algorithms In the Proceedings of 8th Pacific Asia Conference on Knowledge Discovery and Data Mining, Sydney, Australia 2004 3056 3 12

[B39] Nadeau C Bengio Y Inference for the Generalization Error Machine Learning 2003 52 3 239 281 10.1023/A:1024068626366

