# Supplementary material for: Automatic identification of angiogenesis in double stained images of liver tissue
Source: BMC Bioinformatics. 2009 Oct 8;10(Suppl 11):S13. doi: 10.1186/1471-2105-10-S11-S13 (PMC3226185; doi:10.1186/1471-2105-10-S11-S13)
Supplement: Additional file 1 — Virtual Slides. Two virtual slides we used in this study and their technical properties are summarized. [file 1471-2105-10-S11-S13-S1.pdf]

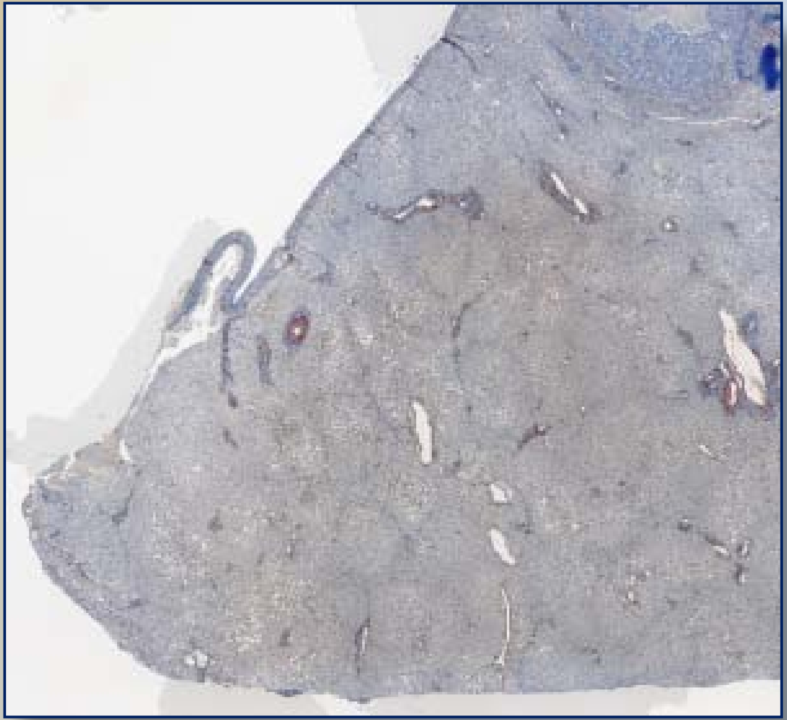

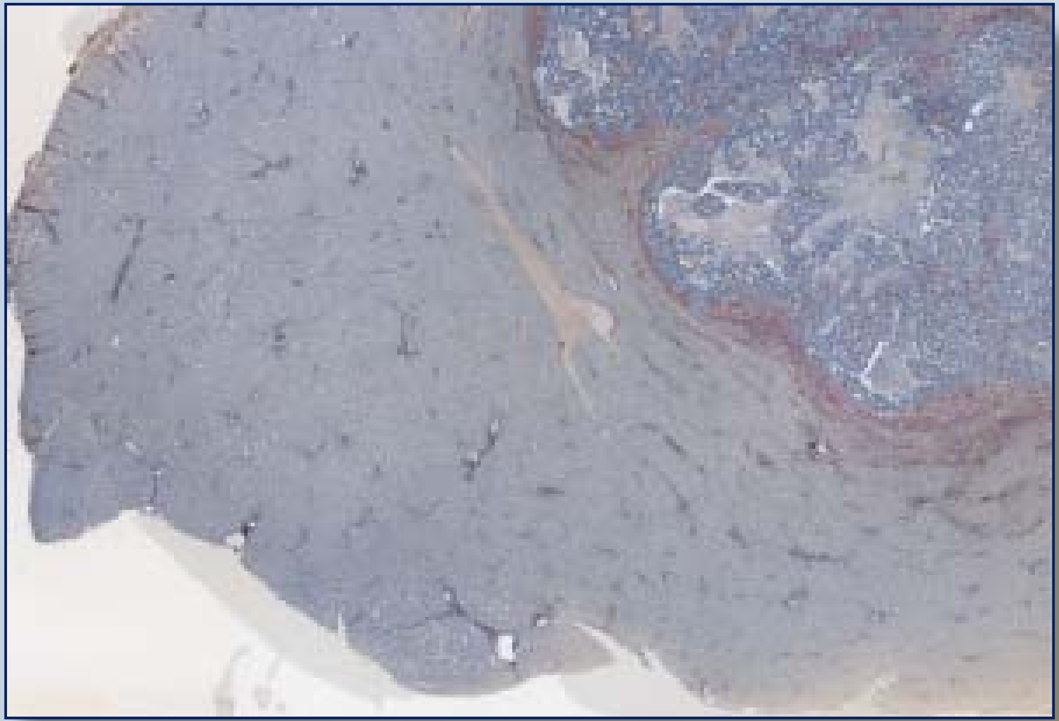

Virtual Slide S_1

- 21943 x 21726 pixels
- Subdivided into 10594 subimages
- Digitalized at 200X
- 0.4667 *μm/pixel*
- Contributed 214 positive and 204 negative subimages

Virtual Slide S_2

- 33912 x 24876 pixels
- Subdivided into 18746 subimages
- Digitalized at 200X
- 0.4667 *μm/pixel*
- Contributed 80 positive and 77 negative subimages

*To keep size of display images consistent, virtual slides are downgraded at different scales .
