# Supplementary figures and images for: Automatic identification of angiogenesis in double stained images of liver tissue
Source: BMC Bioinformatics. 2009 Oct 8;10(Suppl 11):S13. doi: 10.1186/1471-2105-10-S11-S13 (PMC3226185; doi:10.1186/1471-2105-10-S11-S13)

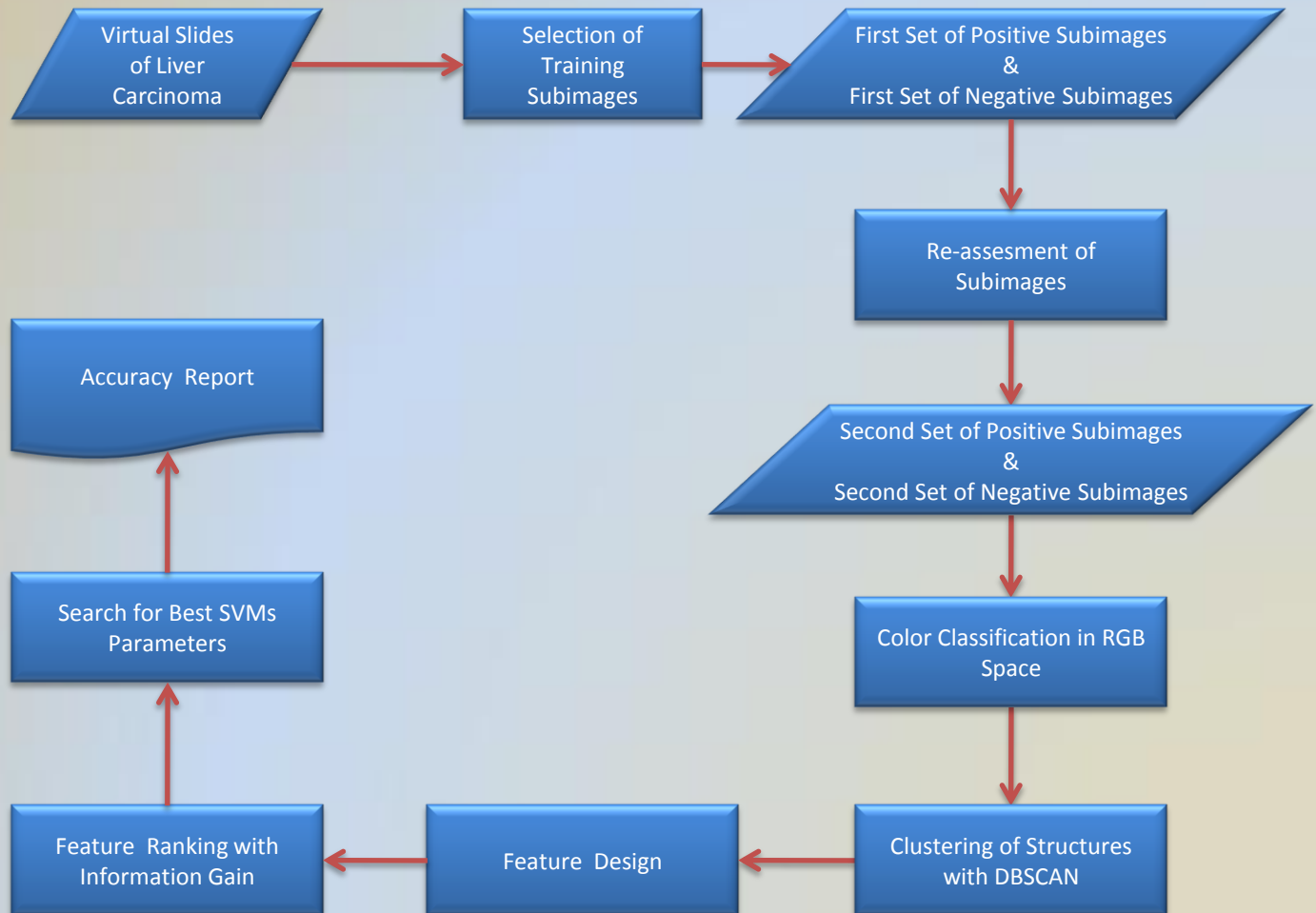

Supplement: Additional file 2 — General Flowchart of the Study. Flowchart of this study is shown. [file 1471-2105-10-S11-S13-S2.pdf]
